# Mesenteric abscess caused by coinfection with Bacillus Calmette-Guérin and *Phialemonium* sp. in chronic granulomatous disease

**DOI:** 10.1016/j.idcr.2022.e01375

**Published:** 2022-01-03

**Authors:** Hanae Miyazawa, Yusuke Matsuda, Seisho Sakai, Katsuhiko Kamei, Taizo Wada

**Affiliations:** a Department of Pediatrics, School of Medicine, Institute of Medical, Pharmaceutical and Health Sciences, Kanazawa University, 13-1 Takaramachi, Kanazawa 920-8641, Japan; b Department of Pediatric Surgery, Kanazawa University Hospital, 13-1 Takaramachi, Kanazawa 920-8641, Japan; c Medical Mycology Research Center, Chiba University, 1-8-1 Inohana, Chuo-ku, Chiba 260-8673, Japan

**Keywords:** Bacillus Calmette-Guérin, Chronic granulomatous disease, Coinfection, Mesenteric abscess, *Phialemonium*

## Abstract

Opportunistic infections are life-threatening conditions in immunocompromised patients including those with primary immunodeficiency. We describe a case of X-linked chronic granulomatous disease presenting with mesenteric abscess caused by a coinfection with Bacillus Calmette-Guérin (BCG) and *Phialemonium* sp. The patient received BCG vaccination at 5 months old. He developed left axillary BCG lymphadenitis at 17 months of age, and 3 months later mesenteric abscess occurred. Concomitant use of rifampicin and itraconazole at 17 months of age might have reduced serum itraconazole concentrations and led to superinfection with *Phialemonium* sp. in our patient, which was susceptible to itraconazole and voriconazole *in vitro*. The patient was successfully treated with a combination of isoniazid, rifampicin, streptomycin, ciprofloxacin, prednisolone, interferon-γ, and an increased dose of itraconazole, followed by hematopoietic stem cell transplantation. Our results suggest that clinician need to be aware of rifampicin drug interactions, and that precise detection and identification of pathogens are essential to appropriate treatment.

## Introduction

Opportunistic infections are life-threatening events in immunocompromised conditions including primary immunodeficiency diseases, which require immediate medical attention. Identification of pathogens from clinical samples is essential to appropriate treatment, but it may be difficult in patients treated with antimicrobial agents as microorganisms grow slowly or not at all under standard laboratory conditions [1]. In addition, coinfection with bacterial and fungal infections are occasionally observed in immunocompromised patients. Chronic granulomatous disease (CGD) is one of the most common primary immunodeficiency that is characterized by a dysfunction in phagocytes to produce reactive oxygen species (ROS). Patients with CGD are susceptible to bacterial, fungal, and mycobacterial infections. In the countries where Bacillus Calmette-Guérin (BCG) vaccination is mandatory in infancy, BCG is often vaccinated before a CGD diagnosis and causes localized or regional BCG infection (*i.e.*, BCG-itis) or disseminated BCG infection (*i.e.*, BCG-osis) [2], [3]. *Phialemonium* species are widely distributed fungi in the environment such as air, soil, industrial water, and sewage [4]. With increasing numbers of immunocompromised individuals, they are recognized as potential pathogens causing severe infections [5].

## Case

**Fig. 1:**
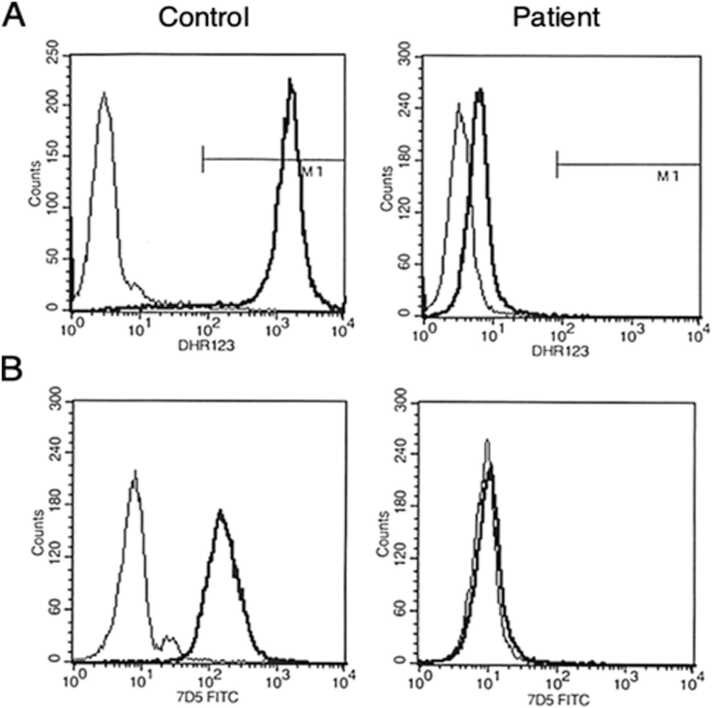
Analysis of dihydrorhodamine-123 (DHR123) and flavocytochrome b558. A. Granulocytes were analyzed using DHR123 as a fluorescent probe before (thin lines) and after (thick lines) stimulation with phorbol myristate acetate. B. Surface expression of flavocytochrome b558, comprised of gp91phox and p22phox, in granulocytes was analyzed by monoclonal antibody 7D5. Thin lines indicate the isotype control antibody and thick lines represent 7D5 antibody.

We report a pediatric case of X-linked CGD (X-CGD) presenting with mesenteric abscess caused by a coinfection with BCG and *Phialemonium*. The patient received BCG vaccination at 5 months old. At 8 months old, he was referred to our hospital because of multiple episodes of pneumonia, cervical lymphadenitis, and subcutaneous abscesses. Dihydrorhodamine-123 analysis showed a marked reduction in his oxidative burst (Fig. 1A). Flow cytometry analysis using monoclonal antibody 7D5 showed a lack of flavocytochrome b558, comprised of gp91phox and p22phox (Fig. 1B). Genetic analysis showed a large deletion mutation from exons 5 to 8 in the *CYBB* gene that encodes gp91phox protein (data not shown). Based on these findings a diagnosis of X-CGD was made, and prophylactic use of trimethoprim-sulfamethoxazole and itraconazole was initiated.

**Fig. 2:**
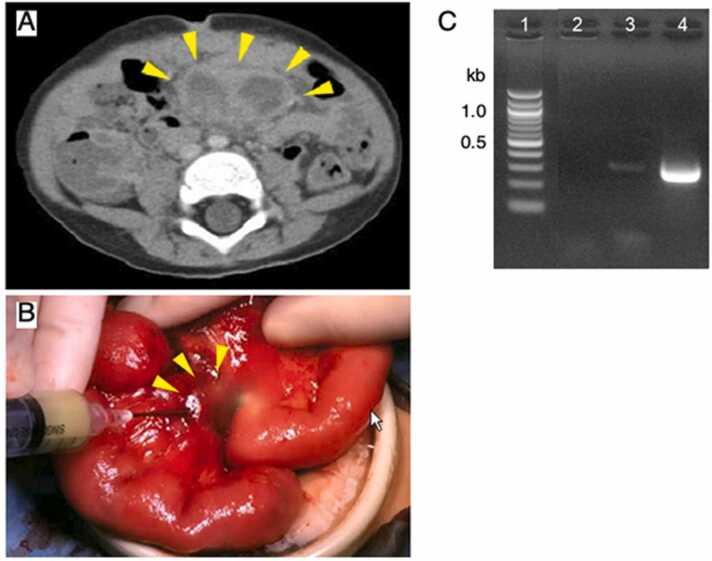
Mesenteric abscess. A. The contrast-enhanced computed tomography image shows an abdominal abscess with a diameter of 4 cm. B. Laparotomic surgery shows a nonpedunculated abscess in the mesentery 40 cm from ligament of Treitz. Approximately 10 mL of pus was aspirated by needle puncture. C. Polymerase chain reaction testing of the aspirated pus shows a 379-base pair fragment, which indicates specific region of difference 16 of the BCG Tokyo strain. Lane 1 is the 100-base pair ladder; lane 2, the negative control; lane 3, the pus from our patient’s mesenteric abscess; and lane 4, the BCG Tokyo strain.

At 17 months old, he developed calcified left axillary lymphadenitis with abscess formation. He subsequently underwent resection of the lymphadenitis, followed by antimycobacterial treatment including isoniazid and rifampicin because of the detection of Mycobacterium tuberculosis complex from the lymphadenitis by polymerase chain reaction. The patient was thought to have regional BCG infection. At 20 months old, he was rehospitalized with recurring fever and an elevated C-reactive protein level (4.2 mg/dL). Computed tomography imaging revealed an abdominal abscess (Fig. 2A). He immediately underwent laparotomic drainage. A nonpedunculated mass with a diameter of 4 cm, which had no connection to the gut lumen, was in the mesentery 40 cm from the ligament of Treitz (Fig. 2B). Approximately 10 mL of pus was aspirated from the mesenteric abscess by needle puncture. A smear of the pus showed acid-fast bacilli, and specific region of difference (RD) 16 of the BCG Tokyo strain was identified by polymerase chain reaction (Fig. 2C), followed by direct sequencing analysis (data not shown). He was diagnosed with BCG-osis. An unidentified fungus was also isolated from the pus, and it was identified as *Phialemonium* species, the most likely *P. curvatum*, by sequencing of nuclear ribosomal internal transcribed spacer and D1/D2 ribosomal DNA [6]. The minimum inhibitory concentrations were of amphotericin B, 0.06 mg/L; itraconazole, ≤ 0.015 mg/L; voriconazole, ≤ 0.015 mg/L; fluconazole, 1 mg/L; flucytosine,> 64 mg/L; miconazole, 0.125 mg/L; and micafungin, 16 mg/L. The patient was treated with a combination of isoniazid, rifampicin, streptomycin, itraconazole, ciprofloxacin, prednisolone, and interferon-γ. We chose itraconazole as an antifungal agent, because coadministration of voriconazole and rifampicin is contraindicated. It is well known that rifampicin is a potent inducer of cytochrome P-450 and significantly reduces serum levels of itraconazole due to enhancement of its metabolism [7]. Therefore, the dose of itraconazole was increased to 40 mg/kg from 5 mg/kg per day. Two months later, the abscess had nearly disappeared. The patient underwent hematopoietic stem cell transplantation (HSCT) with a phenotypically 8/8 HLA-matched unrelated donor at age 3 years. At 17 days and 31 days post-HSCT, he exhibited full donor chimerism. He has been in good health with no evidence of disease for 3 years.

## Discussion

In CGD patients, differences in residual ROS production due to the genotype or the type of inheritance affects the development of severe infections [8]. In X-CGD patients, the mutations in *CYBB* other than missense mutations have fewer ROS production than missense mutations in *CYBB* and would cause more severe infections. However, the development of BCG infections has been reported to be independent of genotype or mutant forms [3], [8], [9]. Under the Japanese vaccination program, children generally receive BCG vaccination before 1 year old. Approximately 67% of CGD patients receive BCG vaccination before a CGD diagnosis [8]. BCG-osis, the most fatal reaction after BCG vaccination, is highly observed in approximately 30% of BCG-vaccinated CGD patients. BCG dissemination is frequently observed in lung, liver, and systemic lymph nodes [2], [3]. The involvement of peritoneum or mesentery like our case is uncommon [9]; however, BCG infection should be suspected in any immunocompromised patients susceptible to mycobacteria who present with persistent fever with inflammation of body tissues.

It has been reported that coinfection, such as bacterial plus fungal infection, is found in less than 10% of biopsy specimens from patients with CGD [10]. To our knowledge, our case is the first description of coinfection with BCG and *Phialemonium. Phialemonium* species include *P. obovatum,* and *P. curvatum*, and *P. dimorphosporum*. They are rarely isolated from clinical samples, but are increasingly recognized to cause invasive human infections, such as peritonitis, endocarditis, osteomyelitis, and cutaneous infections of wounds following burns [4]. Factors predisposed to *Phialemonium* infections were a history of renal or stem cell transplantation, or end-stage kidney disease on hemodialysis with arteriovenous grafts [11], [12], [13]. *P. curvatum* is generally susceptible to voriconazole and posaconazole [5]. Indeed, *in vitro* antifungal susceptibility testing of the isolate showed susceptibility to voriconazole as well as itraconazole in our patient. Concomitant use of rifampicin and itraconazole at 17 months old might have reduced serum itraconazole concentrations, resulting in superinfection with *Phialemonium* in our case.

In summary, we report mesenteric abscess caused by a coinfection with BCG and *Phialemonium* in a patient with CGD. Our results further support the idea that clinician need to be aware of rifampicin drug interactions and that precise detection and identification of pathogens can lead to clinical benefit.

## Funding

This work did not receive any specific grant from funding agencies in the public, commercial, or not-for-profit sectors.

## Ethical approval

All procedures performed in studies involving human participants were in accordance with the 1964 Helsinki declaration and its later amendments.

## Consent

Written informed consent was obtained from the parents for publication of this case report and accompanying images.

## CRediT authorship contribution statement

**Hanae Miyazawa** and **Taizo Wada** wrote and critically revised the manuscript. **Taizo Wada**, **Yusuke Matsuda**, and **Katsuhiko Kamei** analyzed the data and provided a critical paper review. **Seisho Sakai** made clinical contributions. All authors read and approved the final manuscript.

## Conflicts of interest

None to declare.
